# Can the MMPI Predict Adult ADHD? An Approach Using Machine Learning Methods

**DOI:** 10.3390/diagnostics11060976

**Published:** 2021-05-28

**Authors:** Sunhae Kim, Hye-Kyung Lee, Kounseok Lee

**Affiliations:** 1 Department of Psychiatry, Hanyang University Medical Center, Seoul 04763, Korea; sunhk0906@hanyang.ac.kr; 2 Department of Nursing, College of Nursing and Health, Kongju National University, Gongju 32588, Korea; hklee@kongju.ac.kr

**Keywords:** adult ADHD, MMPI-2, screening, detection, machine learning

## Abstract

(1) Background: Adult attention-deficit/hyperactivity disorder (ADHD) symptoms cause various social difficulties due to attention deficit and impulsivity. In addition, in contrast to ADHD in childhood, ADHD in adulthood is difficult to diagnose due to mixed psychopathologies. This study aimed to determine whether it is possible to predict ADHD symptoms in adults using the Minnesota Multiphasic Personality Inventory-2 (MMPI-2) with machine learning (ML) techniques; (2) Methods: Data collected from 5726 college students were analyzed. The MMPI-2-Restructured Form (MMPI-2-RF) was used, and ADHD symptoms in adults were evaluated using the Attention-Deficit/Hyperactivity Disorder Self-Report Scale (ASRS). For statistical analysis, three ML algorithms were used, i.e., K-nearest neighbors (KNN), linear discriminant analysis (LDA), and random forest, with the ASRS evaluation result as the dependent variable and the 50 MMPI-2-RF scales as predictors; (3) Results: When the KNN, LDA, and random forest techniques were applied, the accuracy was 93.1%, 91.2%, and 93.6%, respectively, and the area under the curve (AUC) was 0.722, 0.806, and 0.790, respectively. The AUC of the LDA method was the largest, with an excellent level of diagnostic accuracy; (4) Conclusions: ML using the MMPI-2 in a large group could provide reliable accuracy in screening for adult ADHD.

## 1. Introduction

Attention-deficit/hyperactivity disorder (ADHD) is a neurodevelopmental disorder characterized by inattention, hyperactivity, and impulsivity [1]. Around 50–80% of patients diagnosed with ADHD in childhood report that symptoms persist until adulthood [2,3].

ADHD has been regarded as a childhood-specific disorder for a long time because it is thought that ADHD symptoms improve as children develop [4]; however, long-term follow-up studies report that patients diagnosed with ADHD in childhood meet the diagnostic criteria even as adults [5,6,7,8,9]. Adult ADHD can generally be defined as late adolescents and adults (17 years of age or older). It is a mental disorder that includes a combination of problems, such as inattention, hyperactivity, and impulsiveness, and these problems cause a social functional impairment. The main symptoms of ADHD in adults appear to be carelessness and impulsiveness. Nevertheless, ADHD in adults show marked improvement in hyperactivity symptoms [1,10].

Adults with ADHD may have difficulties in interpersonal relationships, and it is known that they face problems at work, such as the inability to organize and process work efficiently [11,12]. ADHD in adults has a different pattern compared with that of ADHD in children; thus, it is often underdiagnosed in adults and not recognized by clinicians [13]. The main diagnostic features of ADHD are carelessness and hyperactivity/impulsivity; however, in adult ADHD, symptoms such as problems with executive function, emotion regulation, self-concept, self-esteem, and interpersonal relationships are the main features in addition to the main diagnostic characteristics [14,15,16,17,18,19,20,21,22]. Consequently, it is difficult to accurately screen adult ADHD patients only using the diagnostic criteria of the Diagnostic and Statistical Manual of Mental Disorders (DSM) or International Classification of Diseases (ICD) system in the clinical field. Therefore, there is a need for an effective screening tool for adult ADHD [23].

The number of adults diagnosed with ADHD in the United States has steadily increased over the past 20 years [24,25]. The Centers for Disease Control and Prevention (CDC) examined the treatment of adult ADHD from 2002 to 2005 and reported that the percentage of people receiving stimulants for ADHD treatment increased by 90.0% [26], and they were often misused or abused for leisure and not used for therapeutic purposes [27,28,29,30]. Moreover, patients diagnosed with ADHD are given extra time to complete tasks for exams and classes and are provided with special benefits such as lighter workloads, preferred seating, and no penalties for misspellings [31]. Therefore, it is important to identify individuals who falsely report symptoms of ADHD.

Currently, the Adult ADHD Self-Report Scale (ASRS) developed by the World Health Organization can be used to screen for adult ADHD. It is a self-reported scale consisting of 18 items suitable for adulthood based on the Diagnostic and Statistical Manual of Mental Disorders, Fourth Edition (DSM-IV) diagnostic criteria. It has adequate sensitivity (68.7%), excellent specificity (99.5%), and excellent total classification accuracy (97.9%) [32]. However, it is difficult to confirm the reliability of self-reports due to insincere reports.

There have been efforts to use the Minnesota Multiphasic Personality Inventory-2 (MMPI-2) as a detection tool for exaggerated or hidden ADHD [31,33]. The MMPI-2 is one of the most widely used objective personality tests in the world for psychopathology and emotional function evaluation [34,35,36]. Among them, the MMPI-2-Restructured Form (MMPI-2-RF) is a psychometrically improved and more efficient short version that emphasizes the clinical scales reconstructed in MMPI-2 [37]. The MMPI-2 is widely used in psychological evaluation and can simultaneously evaluate response validity during clinical evaluation [31]; thus, it is most commonly used for psychopathological evaluation in medical and forensic fields [38,39,40].

Machine learning (ML) is defined as a computational strategy that automatically determines methods and parameters to arrive at an optimal solution to a problem [41]. A machine learns from data with minimal human intervention, recognizes patterns in data, and proposes to improve diagnostic and prognostic accuracy. This approach appears to be particularly useful in predicting human behavior, including high-risk behavior, and it can be applied to improve the effectiveness and goals of prevention programs and interventions [41]. In comparison with conventional statistical approaches, ML technology has advantages in accuracy and scalability in terms of prediction [42]; thus, several recent studies have applied ML technology to differentiate ADHD from control groups. These studies showed moderate accuracy using linear classifiers [43,44,45,46,47], and it seems that a greater number of more precise studies are needed to predict ADHD through ML.

This study aimed to differentiate risk groups with significant adult ADHD symptoms by applying the latest ML algorithms using the MMPI-2. In order to compensate for the under-assessment of symptoms due to mixed pathologies and self-reports of adult ADHD by using a valid scale that can detect the examinee’s psychopathology exaggeration and clinical scales to identify personality traits related to psychopathology with the MMPI-2, this study aimed to overcome the problem of screening for adult ADHD.

## 2. Materials and Methods

We used part of the dataset of a survey conducted at Kongju National University [48,49,50]. The subjects were informed of the guarantee of anonymity and the use of the survey results for research purposes, and written consent was obtained. The data came from 5806 respondents who completed both the MMPI and ASRS. Data from 5726 respondents were analyzed, excluding the data of 51 subjects who had many omissions and 29 subjects who did not meet the validity criteria of the MMPI-2-RF. This study was approved by the Kongju National University Institutional Review Board (approval No. KNU2015-38, 28 July 2015).

For the MMPI-2-RF, a total of 50 scales that can effectively measure the clinical significance of MMPI-2 items were developed and consisted of 8 validity scales and 42 major scales. The validity criteria for the MMPI were not responding to 30 or more non-response questions, a Variable Response Inconsistency (VRIN) *t*-score of 80 or more, and a True Response Inconsistency (TRIN) *t*-score of 80 or more according to the nonclinical scene guidelines in the MMPI-2 manual [51]. In this study, we used the Korean version of the MMPI-2-RF, and its reliability and validity were verified [52].

The ASRS is a screening tool consisting of 18 questions about the latest frequency of symptoms based on the DSM-IV criteria symptoms of adult ADHD developed by the World Health Organization [4,32]. The ASRS questions are expressed slightly differently from those on the ADHD rating scale for children, and as a screening test with high sensitivity and specificity including the content of ADHD symptoms in adults, the tool has great applicability in clinical and research settings [32].

Each item evaluates the frequency of occurrence of specific symptoms of ADHD over the past 6 months on a 5-point scale, and response options range from 0 to 4 points per question. In our study, the ASRS (+) group was defined as subjects whose total score in the first 9 questions (part 1) or the last 9 questions (part 2) exceeded 21 [53]. 

In this study, the MMPI-2-RF and ASRS were inputted into the ML algorithm. We used three ML classification methods: K-nearest neighbors (KNN), linear discriminant analysis (LDA), and random forest. Of the ML algorithms, K-nearest neighbors (KNN) algorithm is non-parametric classification method, which collects the existing classes and classifies new classes based on the comparison measure [54]. Linear discriminant analysis (LDA) is the correlation between categorical variable and an interrelated variable is considered [55]. Additionally, random forest algorithm is the grouping of tree predictors such that each tree is influenced by the values of a random vector experimented individually and with the same dissemination for all trees in the forest [56]. Outcome variables were analyzed by dividing the subjects into ASRS (+) and ASRS (−) groups, and 50 scales of the MMPI-RF were used as explanatory variables. To assess diagnostic accuracy, we used the area under the curve (AUC), which reflects how good the test is at distinguishing between patients with disease and those without disease. An AUC of 0.5 suggests no discrimination, 0.7–0.8 is considered acceptable, 0.8–0.9 is considered excellent, and more than 0.9 is considered outstanding [57].

Of the total sample, 20% was used as the test dataset, and the remaining 80% were used as the validation dataset; the data were randomly separated into the training dataset and test dataset. To prevent machine learning from overfitting, a validation dataset was added as a verification step. If the model was overfitting, the prediction rate or the error rate would fall, so 20% of the entire dataset was designated as the test dataset and 80% as the validation dataset [58,59]. All statistical analyses were performed using JASP v0.14.1 (Amsterdam, The Netherlands. Released on 17 December 2020) [60]. All *p* values were obtained using a two-sided test, and *p* < 0.05 was considered statistically significant.

## 3. Results

### 3.1. Experimental Results

#### 3.1.1. General Characteristics

A total of 347 subjects (6.0%) were above the cutoff point in the ASRS part 1, and 109 subjects (1.9%) were above the cutoff point in the ASRS part 2. A total of 381 patients (6.6%) were classified into the ASRS (+) group as patients exceeding the cutoff point in part 1 or 2. There were 2851 males (49.8%), and the mean age was 19.8 years (SD = 1.3). There was no significant difference in age between the two groups (*t* = 0.428, *p* = 0.669). The total ASRS score was 4.53 (SD = 0.93) in ASRS (+) group and 4.69 (SD = 1.41) in ASRS (-) group. However, all restructured clinical (RC) scales showed high scores in the ASRS (+) group (Table 1).

#### 3.1.2. Prediction Accuracy of ML Model

Of the 5726 subjects for ML, 3664 subjects were used as the training dataset, 917 subjects were used as the validation dataset, and 1145 subjects were used as the test dataset. In this study, the prediction accuracy of the KNN, LDA, and random forest methods was 93.1%, 91.2%, and 93.6%, respectively (Table 2).

#### 3.1.3. AUC of ML Model

**Figure 1:**
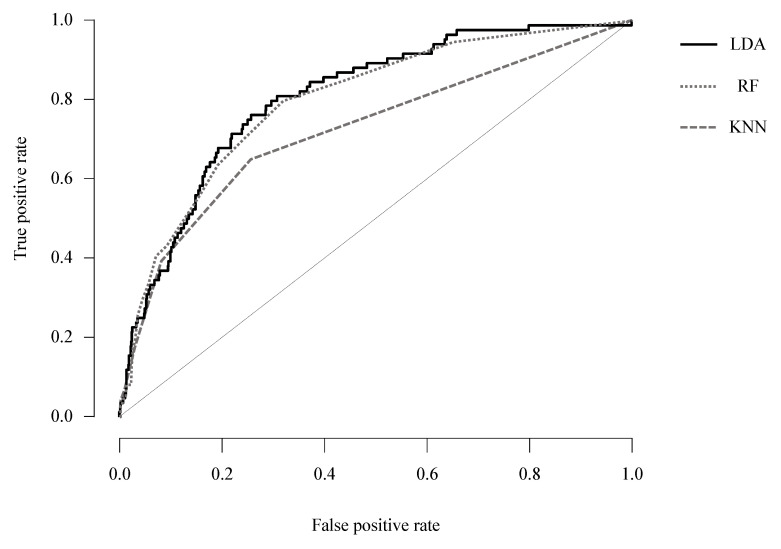
ROC curve plots for each machine learning methods; LDA: linear discriminant analysis; RF: random forest classification; KNN: K-nearest neighbors classification.

The AUC of the KNN, LDA, and random forest methods was 0.722 (recall = 0.931, precision = 0.900), 0.806 (recall = 0.912, precision = 0.899), and 0.790 (recall = 0.936, precision = 0.916), respectively. The AUC of the LDA method was the largest, with an excellent level of diagnostic accuracy, and the rest had an acceptable level of diagnostic accuracy (Table 3, Figure 1).

## 4. Discussion

This study aimed to predict and report adult ADHD symptoms using ML with 50 scales of the MMPI-2-RF, the most commonly used self-report evaluation tool. The three ML algorithms showed high accuracy (91.2–93.6%). Although there were differences according to each method, ADHD symptoms in adults were predicted with an excellent level of accuracy. Therefore, using a common screening tool, the MMPI-2-RF, risk factors related to poor concentration, a symptom of ADHD in adults, may be predicted using ML algorithms.

In our study, most of the clinical scales were high in the ASRS (+) group. This indicates emotional difficulties in adult ADHD, which can be screened using tools that measure existing psychopathology. Although several emotional difficulties may be attributed to concentration disorders and behavioral symptoms, a general increase in psychopathology symptoms is not specific to adult ADHD.

A major difficulty in screening for adult ADHD is that it often has multiple comorbidities and coexistence pathologies [61]. These symptoms of ADHD may be mistaken to be part of other mental disorders requiring clinical attention [62]. Therefore, the ASRS is used as a differential diagnosis tool for various psychopathologies (e.g., substance use disorder) that are mixed with ADHD and thus are difficult to diagnose [32].

In particular, it is important to differentiate the diagnoses of various mental disorders in ADHD including adult ADHD; for this purpose, discrimination studies through ML are being conducted [54,55,56,57]. However, there are various diagnostic tools and methods for ADHD diagnosis, and although ML techniques may be used to diagnose ADHD through neurological tests, a screening tool for adult ADHD diagnosis (ASRS, Conners rating scale, etc.) is still used in the clinical field. Studies that distinguish the symptoms of ADHD using psychological measurement tools are insufficient. As a comprehensive evaluation is required to diagnose adult ADHD, it is possible to initially screen for adult ADHD using efficient screening tools such as the ASRS, which has the advantage of allowing clinicians to discriminate various psychopathological characteristics in advance. A study has demonstrated the clinical use of a screening tool for discriminating autism and ADHD with an accuracy of 82% [63]. Similarly, the accuracy level of the three algorithms in this study was high (91.2–93.6%), and in the case of the LDA technique, an AUC of 0.806 with an accuracy of 91.2% is expected in clinical settings.

However, ML has the disadvantage of not being able to accurately describe the relationship between input and output [64]; thus, it is difficult to precisely determine the complex influence of selected characteristics in classification models. Therefore, if a positive finding is found in the screening test using the ASRS, clinicians should determine whether there is another psychiatric diagnosis, and in adults, it is necessary to confirm the presence of other psychiatric symptoms based on the developmental history [65]. In the case of adult ADHD, concomitant diseases are common, and the evaluation itself is difficult and complex; thus, adult ADHD diagnosis is challenging with the existing criteria, and there is a need to develop specific diagnostic criteria [66].

This study has some limitations. First, the results may not be representative of the entire population as the survey was conducted at one university. In particular, age homogeneity may limit the applicability of the results. Second, the study consisting of a self-reported questionnaire on adult ADHD symptoms in a non-clinical group lacked clinical diagnosis. In addition, there was no information on coexisting diseases or psychiatric treatment history. Considering that this was a retrospective analysis using data in a large university subjects, it was difficult to obtain the relevant information. In the future, the findings will need to be confirmed through follow-up studies that continuously evaluate the symptoms of ADHD in various populations, including clinical evaluation and analysis (factor analysis, principal component analysis, etc.). In addition, this study selected adult ADHD using KNN, LDA, and random forest among machine learning techniques based on clinical theory, but we will suggest extended techniques that use various machine learning classification techniques (vector machine or neural network).

The strength of this study is that it is a large-scale, multi-faceted analysis of the same group of college students. In particular, the limitations of self-report tests were offset using the valid scale of the MMPI-2-RF. In the future, it may be possible to evaluate adult ADHD symptoms in a setting where only the MMPI-2 is used, such as assessment for employment and physical examination for military service.

Thus far, studies that discriminate or classify ADHD through ML algorithms have mostly been based on neurological tests (fMRI, EEG, etc.) [67,68,69]. This study has great significance in that ML was used with the ASRS, which is a screening tool for ADHD, and the MMPI-2, which discriminates psychopathological personality characteristics.

The screening of adult ADHD symptoms is not conclusive with only the MMPI-2, and the use of the MMPI-2-RF makes it possible to obtain valid test results for various aspects of psychopathology. If used together with ML, it will be helpful as an auxiliary tool. In the future, if clinical data are added and analyzed for a wider variety of groups, the possibility of using the MMPI-2 in the evaluation of ADHD symptoms in adults is expected to increase.

## 5. Conclusions

This study demonstrated that ML using the MMPI-2-RF could provide reliable accuracy in classifying and predicting adult ADHD symptoms. This will help clinicians to detect and treat adult ADHD symptoms early in clinical settings.

## Figures and Tables

**Table 1:** The differences between groups.

Dimension	Scale	ASRS (−)			ASRS (+)			*t*	*p*	Cohen’s d
Validity Indicators	VRIN_r	4.19	±	2.37	5.27	±	2.69	−8.51	<0.001	−0.45
	TRIN_r	10.37	±	1.97	11.25	±	2.27	−8.28	<0.001	−0.44
	F_r	3.57	±	3.35	7.59	±	4.64	−21.99	<0.001	−1.17
	Fp_r	2.18	±	2.02	3.86	±	2.88	−15.12	<0.001	−0.80
	Fs	2.15	±	1.94	4.14	±	2.47	−18.90	<0.001	−1.00
	FBS_r	9.11	±	3.48	12.01	±	4.07	−15.50	<0.001	−0.82
	L_r	4.46	±	2.24	3.25	±	2.09	10.24	<0.001	0.54
	K_r	7.24	±	2.67	4.77	±	2.29	17.54	<0.001	0.93
Higher- Order (H-O)	EID	13.99	±	6.60	20.87	±	7.22	−19.55	<0.001	−1.04
	THD	2.55	±	2.70	4.86	±	3.95	−15.61	<0.001	−0.83
	BXD	6.16	±	2.96	8.47	±	3.04	−14.68	<0.001	−0.78
Restructured Clinical (RC)	RCd	7.38	±	5.17	13.54	±	5.40	−22.41	<0.001	−1.19
	RC1	6.62	±	3.93	10.17	±	4.83	−16.75	<0.001	−0.89
	RC2	5.53	±	3.11	6.57	±	3.20	−6.32	<0.001	−0.34
	RC3	4.76	±	2.58	6.69	±	2.81	−13.99	<0.001	−0.74
	RC4	4.03	±	2.60	6.21	±	3.03	−15.60	<0.001	−0.83
	RC6	1.46	±	1.83	2.99	±	2.78	−15.07	<0.001	−0.80
	RC7	7.78	±	4.31	12.59	±	4.47	−20.98	<0.001	−1.11
	RC8	3.18	±	2.59	5.72	±	3.22	−18.17	<0.001	−0.96
	RC9	11.28	±	4.61	14.71	±	4.01	−14.19	<0.001	−0.75
Content, Clinical Subscale	MLS	3.02	±	1.60	3.97	±	1.77	−11.08	<0.001	−0.59
	GIC	0.64	±	1.04	1.21	±	1.36	−9.99	<0.001	−0.53
	HPC	1.31	±	1.37	2.25	±	1.74	−12.71	<0.001	−0.67
	NUC	2.99	±	1.69	4.02	±	1.74	−11.56	<0.001	−0.61
	COG	3.06	±	2.13	5.68	±	2.16	−23.18	<0.001	−1.23
	SUI	0.29	±	0.74	0.82	±	1.25	−12.72	<0.001	−0.67
	HLP	1.00	±	1.07	1.82	±	1.28	−14.23	<0.001	−0.76
	SFD	1.41	±	1.26	2.45	±	1.28	−15.54	<0.001	−0.82
	NFC	4.01	±	2.16	5.73	±	1.98	−15.13	<0.001	−0.80
	STW	2.76	±	1.72	4.20	±	1.76	−15.77	<0.001	−0.84
	AXY	0.42	±	0.82	1.16	±	1.25	−16.50	<0.001	−0.88
	ANP	2.37	±	1.64	3.78	±	1.75	−16.07	<0.001	−0.85
	BRF	2.00	±	1.55	2.73	±	1.77	−8.75	<0.001	−0.46
	MSF	3.97	±	2.31	4.03	±	2.32	−0.45	0.66	−0.02
	JCP	0.83	±	1.07	1.48	±	1.39	−11.35	<0.001	−0.60
	SUB	0.70	±	0.95	1.16	±	1.32	−8.79	<0.001	−0.47
	AGG	2.51	±	1.85	3.90	±	1.99	−14.19	<0.001	−0.75
	ACT	2.36	±	1.74	3.61	±	1.73	−13.57	<0.001	−0.72
	FML	2.07	±	1.85	3.73	±	2.22	−16.72	<0.001	−0.89
	IPP	4.37	±	2.18	4.50	±	2.16	−1.12	0.26	−0.06
	SAV	4.16	±	2.58	4.54	±	2.67	−2.73	0.01	−0.15
	SHY	3.33	±	2.01	4.27	±	1.92	−8.91	<0.001	−0.47
	DSF	0.78	±	1.12	1.37	±	1.46	−9.61	<0.001	−0.51
	AES	2.94	±	1.69	3.12	±	1.76	−2.04	0.04	−0.11
	MEC	2.22	±	1.81	2.41	±	1.88	−1.95	0.05	−0.10
Personality Psychopathology Five (PSY-5)	AGGR_r	7.85	±	3.23	8.56	±	3.14	−4.16	<0.001	−0.22
	PSYC_r	3.07	±	2.79	5.60	±	3.89	−16.58	<0.001	−0.88
	DISC_r	5.73	±	2.54	7.16	±	2.67	−10.61	<0.001	−0.56
	NEGE_r	7.88	±	3.87	11.43	±	3.72	−17.36	<0.001	−0.92
	INTR_r	8.06	±	3.82	8.26	±	3.74	−1.01	0.31	−0.05

ASRS (+): Adult ADHD symptoms group; the subjects whose total score of part 1 or part 2 exceeded 21; values are presented as mean ± SD; ASRS (−): control group. df: 5724.00.

**Table 2:** Accuracy in each predictive model.

	Trees or Nearest Neighbors	Validation Accuracy	Test Accuracy	OOB Accuracy
K-Nearest Neighbors Classification	7	0.927	0.931	
Linear Discriminant Analysis			0.912	
Random Forest Classification	40	0.944	0.936	0.078

The random forest models are optimized with respect to the out-of-bag accuracy. The KNN model is optimized with respect to the validation set accuracy.

**Table 3:** Total average evaluation metrics in each predictive model.

	Precision	Recall	F1 Score	AUC
K-Nearest Neighbors Classification	0.900	0.931	0.909	0.722
Linear Discriminant Analysis	0.899	0.912	0.905	0.806
Random Forest Classification	0.916	0.936	0.909	0.790

Area under curve (AUC) is calculated for every class against all other classes.

## Data Availability

The data presented in this study are available on request from the corresponding author. The data are not publicly available due to ethical restrictions.
